# Psychological Wellbeing of Diabetic Individuals, Prediabetics, and Non-diabetics: A Population-Based Study in Saudi Arabia

**DOI:** 10.3389/fpsyg.2022.863861

**Published:** 2022-06-13

**Authors:** Khaled K. Aldossari, Mamdouh M. Shubair, Sameer H. Al-Ghamdi, Abdulrahman A. Alduraywish, Alhanouf Abdullah Almeshari, Abdullah A. Alrasheed, Raed Aldahash, Khadijah Angawi, Anood Gaissi, Hana Abdullah Alhumud, Ashraf El-Metwally

**Affiliations:** ^1^Family and Community Medicine Department, College of Medicine, Prince Sattam Bin Abdulaziz University, Al-Kharj, Saudi Arabia; ^2^School of Health Sciences, University of Northern British Columbia, Prince George, BC, Canada; ^3^Internal Medicine Department, Jouf University, Sakaka, Saudi Arabia; ^4^Health Cluster, Ministry of Health, Riyadh, Saudi Arabia; ^5^Family and Community Medicine Department, College of Medicine, King Saud University, Riyadh, Saudi Arabia; ^6^King Saud University Medical City, King Saud University, Riyadh, Saudi Arabia; ^7^Department of Medicine, Ministry of National Guard Health Affairs, King Abdullah International Medical Research Center, King Saud bin Abdulaziz for Health Science, Riyadh, Saudi Arabia; ^8^Department of Health Services and Hospital Administration, Faculty of Economics and Administration, King Abdulaziz University, Jeddah, Saudi Arabia; ^9^Research and Education Department, Saudi National Institute for Health Research, Riyadh, Saudi Arabia; ^10^College of Public Health and Health Informatics, King Saud Bin Abdulaziz University for Health Sciences, Riyadh, Saudi Arabia

**Keywords:** diabetes mellitus, prediabetics, psychological wellbeing, GHQ, Saudi Arabia

## Abstract

**Background:**

The increased burden of diabetes affects the quality of life, including psychosocial problems. The study aims to compare the psychological well-being of individuals who are prediabetic, diabetic, or non-diabetic.

**Methods:**

A cross-sectional exploratory study was conducted from January to June 2016 (*n* = 1,019) in Al Kharj, Saudi Arabia. After consent and questionnaires were filled out, trained staff took blood samples followed by anthropometry. Chi-squared tests, one-way ANOVA, and multiple linear regression analyses were conducted to examine the association between diabetes classes defined by HbA1c cut-off levels set by the American Diabetes Association (three categories), individual items, and total score in general health questionnaire (GHQ). An ROC curve was plotted for the total GHQ-12 score against HbA1c.

**Findings:**

The mean GHQ score for psychological distress was significantly higher (F = 6.569, *P* = 0.038) in the diabetics (mean = 14.7) and the prediabetics (12.4) than in the non-diabetics (10.71). Four out of six positive GHQ items and three out of six negative GHQ items significantly differed among the three classes of diabetes. The adjusted multivariate analysis revealed that people with diabetes were most likely to report psychological distress compared to non-diabetics (unstandardized beta = 2.414; *P* = 0.037). The AUC examining the relationship between HBA1c and GHQ scores showed a moderate but statistically insignificant sensitivity/specificity of 0.643 (*P* = 0.23).

**Conclusion:**

This study demonstrates that psychological wellbeing is substantially poorer among diabetic or prediabetic individuals than non-diabetic individuals. Future longitudinal studies are required to examine a plausible causal relationship between diabetes/prediabetes and psychological distress.

## Introduction

A significant global public health concern, diabetes is the most common chronic multifactorial condition affecting individuals of all age groups. The World Health Organization (WHO) estimated that between 1980 and 2014, the rate of diabetes doubled (from 4.7 to 8.5%) and predicted that if current trends continue to progress in the same manner, the number of cases will reach 700 million by 2045 (WHO, 2018; Pradhan et al., 2020). Currently, 463 million cases of diabetes are reported globally (IDF, 2019). Type 2 diabetes comprises most patients with diabetes, which is caused by inefficiency of insulin and is related mainly to environmental factors like dietary intake and physical inactivity (IDF, 2019).

Prediabetes, an intermediate condition of diabetes with blood glucose level above normal or at borderline but below the diabetic threshold, is an early marker indicating a high risk for developing diabetes (Bansal, 2015). Therefore, screening for prediabetes is recommended as lifestyle modification can help reduce or prevent the progression of diabetes by 40–70% (Katon, 2008; Bansal, 2015).

Because of the current high prevalence of diabetes and increasing trends of diabetes in the future, it is imposing a substantial burden on the healthcare system and the individuals' quality of life. Literature related to diabetes is replete in addressing the effect of diabetes on cardiovascular diseases, renal diseases, retinopathy, foot ulcer, sexual dysfunction, and depression (Trikkalinou et al., 2017). However, general health related to functional psychiatric disorders, including social dysfunction, anxiety, confidence, and other related factors, is not well reported. Few studies have reported the quality of life of diabetics in terms of sleep, social support, and depression (Semenkovich et al., 2015; Trikkalinou et al., 2017). The increasing prevalence of diabetes affects the quality of life with more psychosocial problems. Thus, there has been an increasing need to assess psychosocial and mental health among patients with life-long chronic diseases in the last decade, which has become of utmost importance. This is important in identifying important psychological aspects that are affected most. It can also help modify the intervention and treatment needs to improve and manage the condition and target the underlying psychological issues (Trikkalinou et al., 2017). Globally, different studies have used different psychometric tools for assessing the quality of life among diabetic patients and reported high anxiety and depression levels compared to non-diabetic people or individuals not yet diagnosed with diabetes (Das-Munshi et al., 2007; Al-Aboudi et al., 2015).

In Saudi Arabia, diabetes is also one of the significant contemporary chronic conditions affecting children, young adults, and the elderly. Recent studies have reported the prevalence of diabetes and prediabetes among women as 3.8 and 18.8% and 9.2 and 27.6% among men, respectively (Aldossari et al., 2018; Al-Zahrani et al., 2019). In comparison, other community-based studies reported the age- and sex-standardized prevalence of prediabetes as 9% among adults and prevalence of diabetes as 12.1% (Bahijri et al., 2016). Many tools are available for assessing individuals' mental and psychological wellbeing. However, GHQ is considered a very reliable and standard assessment tool for psychological disorders in primary healthcare settings (Montazeri et al., 2003; Jackson, 2007). Moreover, quality of life has been assessed using the Heath Related Quality of Life (HRQoL) tool in Saudi Arabia (Al-Aboudi et al., 2015, 2016). However, the general health questionnaire (GHQ) has not been used previously to evaluate psychological disorder and strain scores of diabetic and prediabetic patients. The GHQ was reported as an efficient and validated tool among the Saudi population for evaluating general health (El-Metwally et al., 2018). As this questionnaire tool is very straightforward and takes only 10 min to complete, it makes it a very efficient and unique self-reported questionnaire to use in an outpatient setting (Montazeri et al., 2003). We aim to compare the psychological wellbeing of individuals with prediabetes to that of individuals with diabetes using the validated GHQ tool in this study.

## Materials and Methods

### Study Design and Setting

A cross-sectional exploratory population-based study was conducted, with data drawn from the general population in Al-Kharj city from January 2016 to the end of June 2016.

### Inclusion and Exclusion Criteria

The selection criteria included Saudi citizens with type 2 diabetes, 18 years of age or older, based on their eligibility as well as willingness to participate in the study. On the other hand, the exclusion criteria were non-Saudi Arabians, patients with type 1 diabetes, younger than 18 years, and individuals not willing to give and sign the informed consent form.

### Sample Size

A sample size of with a total of 1,200 participants was included with a response rate of 85%. Sample size calculation was based on the prevalence of diabetes in the general population in Saudi Arabia, which is 14.4%, while the prevalence of depression among patients with T2DM is 37% (WHO, 2016; Alhunayni et al., 2020). Using a confidence level of 95% and a power of 80%, the sample size needed was estimated to be 600.

### Sampling Technique

A multi-stage sampling method was utilized. A list of all clusters was made, and the investigators drew a random number of clusters to be included in the study. Samples from 21 government and 11 private institutes were selected through a cluster sampling technique. The total population of the institutions was divided into groups called clusters after acquiring a list of participants in each institute nominated. Samples of the respondents were then selected by simple random sampling from each of the groups (cluster).

### Instruments

For data collection, a structured and self-administered questionnaire was used. The questionnaire had multiple sections. Data such as age, education, employment, marital status, and diabetes status were recorded. For the assessment of psychological wellbeing, a self-reported and validated GHQ-12 for the Arabic population was used (Daradkeh et al., 2001). Each item comprises a 4-point scale (0, less than usual; 1, no more than usual; 2, rather more than usual; 3 or more, much more than usual) (Abubakar and Fischer, 2012).

### Blood Sampling and Anthropometry

A trained phlebotomist took blood samples from all the participants to calculate HbA1c measured at baseline. After following all aseptic procedures, a blood sample was taken from all the participants. A test tube with a purple top is used for blood collection for HbA1c, and 3 cc blood was collected from each participant. A unique ID is given to each participant, and the specimen was labeled using a specific ID. Tubes were gently rolled to avoid clotting, and then each tube was placed in a roller mixer. The samples (within 1–2 h) were then taken to the central laboratory in a container with ice. The complete blood sampling procedure has already been defined previously (Aldossari et al., 2018).

Trained staff also took the anthropometric measurements, including weight, height, and waist circumference. Height was taken in inches without shoes in an upright position using a standard scale. Weight was measured in kg in light clothes and without shoes, while waist circumference was measured in cm using a standard measuring tape.

### Operational Definitions

Diabetes mellitus was defined as fasting plasma glucose (FPG) ≥ 126 mg/dl or HbA1c cut-off level of ≥6.5%, or as defined by the American Diabetes Association (ADA) criteria having a history of diabetes. Prediabetes was defined as FPG 100 125 mg/dl or using the HbA1c cut-off level of 5.7–6.4 % according to ADA 2020 (American Diabetes Association, 2020).

In this study, we used HbA1c to define diabetes and prediabetes (as it does not require fasting), and we can collect blood at any time of the day, keeping in mind the convenience of the participants and achieving a large sample size. This is a standard method used for diagnosing diabetes in government hospitals in Saudi Arabia. Furthermore, in this study, we also included participants who were diagnosed with diabetes.

### Statistical Analysis

Data were analyzed using SPSS version 26.0 for Windows (IBM Corp., Armonk, NY, United States). There were six positive items (GHQ-1 to GHQ-6) and six negative items (GHQ-7 to GHQ-12) to assess positive and negative mental health in the GHQ-12 questionnaire. The association of diabetic status (categorical variable: diabetic vs. prediabetic vs. non-diabetic) was compared across all the 12 GHQ variables (GHQ-1 to GHQ-6 positive items and GHQ-7 to GHQ-12 negative items). We conducted a chi-squared (*X*^2^) test for each of the GHQ items against diabetic status. We conducted twelve (*n* = 12) cross-tabulation (*X*^2^) tests to assess the association between each of the 12 GHQ items and diabetic status. We also used a one-way ANOVA model to compare the mean GHQ score across the three groups: non-diabetic, prediabetic, and diabetic. In the one way ANOVA model, Tukey's *post-hoc* multiple comparison tests were performed (the three diabetes groups were compared against each other). A multiple linear regression model was used to examine the association between diabetic classes (three categories) and total GHQ score (outcome).

## Results

Results related to the description of the population and prevalence of diabetes, prediabetes, and general health of the population were published previously (Aldossari et al., 2018; El-Metwally et al., 2018).

We conducted twelve (*n* = 12) cross-tabulation (*X*^2^) to assess the association between each of the 12 GHQ items to diabetic status. The summarized results are presented in Table 1.

**Table 1:** Comparison among diabetic, prediabetic, and non-diabetic subjects according to positive and negative general health questionnaire (GHQ)-12 items in the Al Kharj study (*n* = 1,016).

**GHQ**	**Diabetes (*n* = 44)**	**Pre-diabetes (*n* = 230)**	**No-diabetes (*n* = 741)**	***P*-value**
**Positive items**				
GHQ1: Have you recently been able to concentrate on whatever you're doing?				0.339
0	5 (5.3%)	23 (24.5%)	66 (70.2%)	
1	35 (4.9%)	156 (22.0%)	519 (73.1%)	
2	4 (2.1%)	45 (23.2%)	145 (74.7%)	
3	0 (0.0%)	7 (38.9%)	11 (61.1%)	
GHQ2: Have you recently felt that you were playing a useful part in things?				0.600
0	8 (4.1%)	45 (23.2%)	141 (72.7%)	
1	33 (4.6%)	164 (22.9%)	520 (72.5%)	
2	3 (3.2%)	22 (23.4%)	69 (73.4%)	
3	0 (0.0%)	0 (0.0%)	11 (100.0%)	
GHQ3: Have you recently been feeling reasonably happy, all things considered?				0.031
0	3 (2.6%)	24 (20.5%)	90 (76.9%)	
1	36 (4.7%)	176 (22.9%)	555 (72.4%)	
2	4 (3.4%)	29 (24.4%)	86 (72.3%)	
3	1 (7.7%)	2 (15.4%)	10 (76.9%)	
GHQ4: Have you recently felt capable of making decisions about things?				0.047
0	5 (3.0%)	34 (20.7%)	125 (76.2%)	
1	32 (5.0%)	151 (23.4%)	463 (71.7%)	
2	5 (2.7%)	39 (21.3%)	139 (76.0%)	
3	2 (10.0%)	6 (30.0%)	12 (60.0%)	
GHQ5: Have you recently been able to enjoy your normal day-to-day activities?				0.393
0	7 (5.7%)	30 (24.4%)	86 (69.9%)	
1	30 (4.7%)	147 (23.0%)	461 (72.3%)	
2	7 (3.1%)	51 (22.6%)	168 (74.3%)	
3	0 (0.0%)	3 (10.3%)	26 (89.7%)	
GHQ6: Have you recently been able to face up to problems?				0.036
0	6 (3.8%)	36 (22.9%)	115 (73.2%)	
1	31 (4.5%)	161 (23.2%)	501 (72.3%)	
2	6 (4.1%)	28 (18.9%)	114 (77.0%)	
3	1 (5.9%)	5 (29.4%)	11 (64.7%)	
**Negative items**				
GHQ7: Have you recently felt constantly under strain?				0.085
0	8 (3.8%)	39 (18.5%)	164 (77.7%)	
1	13 (4.7%)	56 (20.1%)	210 (75.3%)	
2	13 (3.4%)	92 (24.1%)	276 (72.4%)	
3	9 (6.3%)	43 (30.1%)	91 (63.6%)	
GHQ8: Have you recently felt you couldn't overcome your difficulties?				0.164
0	18 (4.6%)	84 (21.5%)	288 (73.8%)	
1	4 (1.6%)	54 (21.5%)	193 (76.9%)	
2	21 (6.2%)	83 (24.3%)	237 (69.5%)	
3	1 (3.2%)	8 (25.8%)	22 (71.0%)	
GHQ9: Have you recently lost much sleep over worry?				0.056
0	18 (5.6%)	73 (22.8%)	229 (71.6%)	
1	11 (4.2%)	61 (23.6%)	187 (72.2%)	
2	12 (3.8%)	73 (23.2%)	229 (72.9%)	
3	3 (2.5%)	23 (19.0%)	95 (78.5%)	
GHQ10: Have you recently been feeling unhappy or depressed?				0.030
0	23 (4.3%)	102 (19.2%)	407 (76.5%)	
1	6 (2.8%)	51 (23.7%)	158 (73.5%)	
2	14 (6.3%)	64 (28.6%)	146 (65.2%)	
3	1 (2.3%)	13 (30.2%)	29 (67.4%)	
GHQ11: Have you recently been losing confidence in yourself?				0.017
0	31 (4.7%)	136 (20.5%)	496 (74.8%)	
1	3 (2.1%)	33 (23.1%)	107 (74.8%)	
2	9 (5.0%)	48 (26.5%)	124 (68.5%)	
3	1 (3.7%)	13 (48.1%)	13 (48.1%)	
GHQ12: Have you recently been thinking of yourself as a worthless person?				0.039
0	33 (4.6%)	153 (21.2%)	536 (74.2%)	
1	2 (1.6%)	30 (23.6%)	95 (74.8%)	
2	7 (5.1%)	36 (26.1%)	95 (68.8%)	
3	2 (7.7%)	11 (42.3%)	13 (50.0%)	

*0 (better than usual or > usual).*

*1 (same usual or as usual).*

*2 (worse than usual or less than usual or < usual).*

*3 (much worse than usual or much less than usual or < < usual)*.

### Positive GHQ 12 Item Results

Compared to the prediabetic and diabetic people, the non-diabetic individuals were “better than usual” in being “able to concentrate on whatever they are doing”. However, this result was not statistically significant, as observed by the chi-squared test: (*X*^2^) = 6.806, *P* = 0.339 (Table 1). Regarding GHQ-2, there was no difference among the three groups. When asked whether they have “recently been feeling reasonably happy” (GHQ-3), the data showed that the non-diabetic individuals indicated that they were significantly “better than usual or the same as usual” regarding this positive feeling/item, in comparison with the prediabetic and diabetic individuals. The chi-squared test was (*X*^2^) = 12.717, *P* = 0.03. In terms of whether someone has “recently felt capable of making decisions about things”, the prediabetic and diabetic people were less likely to feel “better than usual or same as usual” than the non-diabetics. The proportion of prediabetics (30%) and diabetics (10%) was higher in what they expressed as “much worse than usual/much less than usual”. The chi-squared test was (*X*^2^) = 15.859, *P* = 0.047. The GHQ-5 results did not show any statistical significance among the three diabetic groups when asked “have you recently been able to enjoy your normal day-to-day activities”. When asked whether they have “recently been able to face up to problems”, the proportion of non-diabetic people who reported “better than usual or same as usual” was higher than that of the prediabetic and diabetic people. There was a statistically significant result in the chi-squared test: (*X*^2^) = 12.146, *P* = 0.036.

### Negative GHQ 12 Item Results

The GHQ-7 and GHQ-8 results did not show any statistical significance among the three diabetic groups when asked “have you recently felt constantly under strain” and “have you recently felt you couldn't overcome your difficulties”.

There were marginally statistically significant differences among the three diabetic groups when asked “have you recently lost much sleep over worry”. There was a notably higher proportion of the prediabetic individuals (than the diabetic persons) who indicated “worse than usual/or much worse than usual”. The chi-squared test was (*X*^2^) = 13.853, *P* = 0.056. For, GHQ-10, which is “recently been feeling unhappy or depressed”, the prediabetic individuals particularly indicated that they feel “worse than usual” (28.6%) or “much worse than usual” (30.2%) compared with a lesser proportion of the diabetic individuals (6.3% felt “worse than usual” and 2.3% felt “much worse than usual”). The chi-squared test was (*X*^2^) = 13.933, *P* = 0.03. GHQ-11 showed significant findings pertaining to the diabetic and prediabetic individuals reporting significantly higher proportions/responses to the question “have you recently been losing confidence in yourself” in the “negative categories” (worse than usual: 5% for the diabetics and 26.5% for the prediabetics). The chi-squared test was (*X*^2^) = 15.449, *P* = 0.017. Also, GHQ-12 showed significant findings pertaining to the diabetic and prediabetic individuals reporting significantly higher proportions/responses to the question “have you recently been thinking of yourself as a worthless person” in the “negative categories”: (“worse than usual”: 5.1% for the diabetics and 26.1% for the prediabetics); “much worse than usual”: 7.7% for the diabetics and 42.3% for the prediabetics). The chi-squared test was (*X*^2^) = 11.459, *P* = 0.039 (Table 1).

### GHQ Score and Diabetic Class Categorical Variable (One-Way ANOVA)

The total GHQ score (mean = 12.6; SD = 5.26), as previously reported, was used (El-Metwally et al., 2018) to compare the three diabetic class categories: non-diabetic, prediabetic, and diabetic subjects. Overall, the one-way ANOVA model was statistically significant (F = 6.569, d = 2, *P* = 0.038).

A comparison between the mean total GHQ score and the three diabetic class categories is shown in Table 2.

**Table 2:** Mean difference in total GHQ-12 score across diabetic class categories.

		***n***	**Mean**	**Std. deviation**	**Std. error**
0 non-diabetic		733	10.71	5.12	0.189
1 pre-diabetic		227	12.41	5.23	0.366
2 diabetic		43	14.69	5.39	0.821
Total		1,003	12.60	5.26	0.165
Model	Fixed effects			5.224	0.165
	Random effects				0.267

### Findings of Tukey's *post-hoc* Analyses

The first *post-hoc* analysis showed that compared to the non-diabetic group (reference category), the diabetic group was statistically significant (mean difference 2.547, *P* = 0.027). The 95% confidence interval (CI) was from 2.148 to 5.381. The second *post-hoc* analysis showed that the prediabetic group was also statistically significant when compared to the non-diabetic group (mean difference 1.185, *P* = 0.042). The 95% CI was from 0.97 to 2.36.

### Findings of Multiple Linear Regression Analyses

Using the entire sample (*n* = 1,018) and after adjusting for sociodemographic variables, the first multiple regression analysis was conducted. It was found that higher psychological distress (as evidenced by higher total GHQ score) was significantly and positively associated with the diabetic class (i.e., in more diabetic and prediabetic individuals). The unstandardized beta regression coefficient was = 2.414 (*P* = 0.037). Being diabetic has 2.4 times greater risk of psychological distress. Women were most likely to have greater psychological distress than men (unstandardized beta = 1.309, *P* = 0.048). Job status, whether unemployed or civilian (civil worker) is significantly associated with higher psychological distress (unstandardized beta = 1.416, *P* = 0.045), as shown in Table 3.

**Table 3:** Multiple linear regression model regressing total GHQ score on diabetic class and other sociodemographic factors (*n* = 1,018).

**Total GHQ score**	**Unstandardized beta (B)**	**S.E. of B**	**Sig**.	**Standardized B**	**95% CI for odds ratio**	
					**Lower**	**Upper**
Diabetic class(binary variable: diabetic vs. non-diabetic individuals)	2.414	0.331	0.037	2.135	2.017	3.461
Age	0.018	0.028	0.516	0.030	0.037	0.074
Gender (female)	1.309	0.465	0.048	1.22	0.397	2.221
Marital status (single/not married)	−0.484	0.443	0.275	−0.045	−1.354	0.386
Job (not working; or civilian)	1.416	0.413	0.045	0.043	1.214	3.402

In the second multiple regression analysis, we only selected individuals who were diabetic (*n* = 45). After adjusting for other variables (sociodemographic), higher total GHQ score (reflecting higher psychological distress) was significantly and positively associated with hypertension status (unstandardized beta regression coefficient = 2.342; *P* = 0.043). “Presence of chronic disease” (either hypertension or dyslipidemia, or both) was also significantly and positively associated with higher psychological distress (unstandardized beta regression coefficient = 4.016; *P* = 0.021). Furthermore, women were more likely to have significantly higher psychological distress (unstandardized beta regression coefficient = 2.51, *P* = 0.028). In terms of employment status, those who were not working (unemployed) or civilian workers were also more significantly susceptible to higher psychological distress (unstandardized beta regression coefficient = 4.723, *P* = 0.039), as evident in Table 4.

**Table 4:** Multiple linear regression model regressing total GHQ score on hypertension status, HBA1c, and other sociodemographic and diabetes risk factors in diabetic individuals (*n* = 45).

**Total GHQ score**	**Unstandardized beta (B)**	**S.E. of B**	**Sig**.	**Standardized B**	**95% CI for odds ratio**	
					**Lower**	**Upper**
Hypertension status	2.342	0.294	0.043	1.186	2.114	3.611
Age	0.021	0.090	0.816	0.044	0.205	0.163
Gender (female)	2.510	0.915	0.028	1.22	1.887	3.691
Marital status (single/not married)	0.123	2.117	0.954	0.010	−4.190	4.435
Education level attainment	0.383	0.808	0.658	0.089	−1.263	2.029
Job (not working; or civilian)	4.723	3.477	0.039	0.234	2.860	5.806
Body Mass Index (BMI)	0.016	0.156	0.918	0.020	−0.302	0.334
Cholesterol level	−0.252	1.165	0.830	−0.052	−2.625	2.122
Smoking status	−2.893	1.563	0.073	−0.340	−6.076	0.291
Waist circumference	0.017	0.033	0.623	0.094	−0.051	0.085
HbA1c	0.581	0.439	0.195	0.231	−0.314	1.476
Presence of chronic disease (hypertension or dyslipidemia)	4.016	3.104	0.021	0.364	2.859	5.244

### Receiver Operating Characteristic (ROC) Curve Analysis

An ROC curve analysis was performed, and the area under the curve was reported. This technique plots a sensitivity (on the Y-axis) against the false positive rate (1-specificity) on the X-axis of a plot. The area under the curve (AUC) normally ranges from 0.5 for models with no discrimination ability to 1 for models with ideal discrimination ability (Kim et al., 2013). Generally speaking, an AUC of 0.5 recommends no discrimination (i.e., ability to diagnose patients with and without the disease), while 0.7–0.8 is suggested as acceptable, 0.8–0.9 is excellent, and more than 0.9 is considered outstanding (Park et al., 2004).

**Figure 1:**
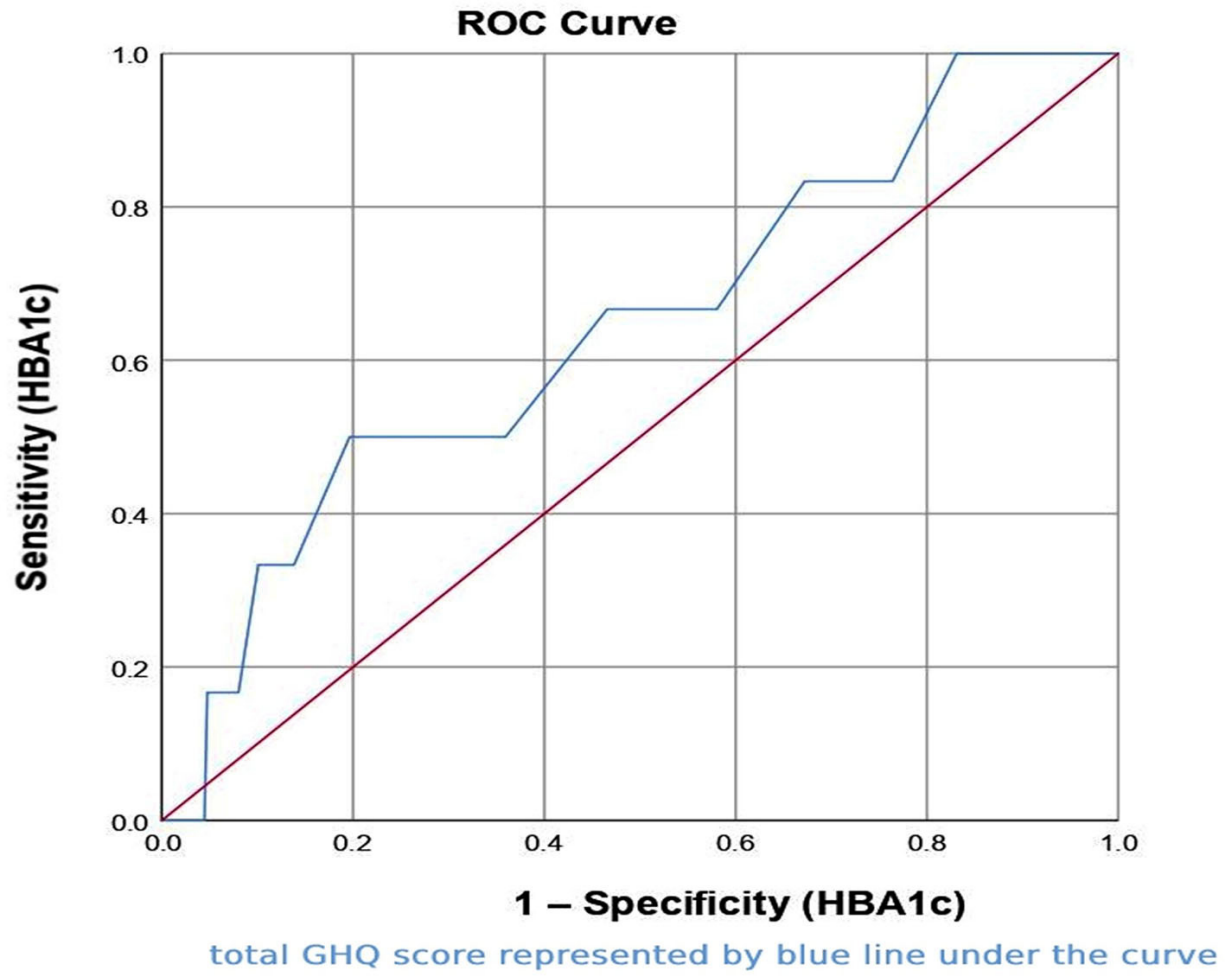
Receiver operating characteristic (ROC) curve for total general health questionnaire (GHQ)-12 score against HbA1c (as the test result variable).

We used the HbA1c continuous variable as the test result (independent) variable. Larger values of the test result variable indicate more substantial evidence of the actual positive state. The AUC examining the relationship between total GHQ score (Figure 1) showed a moderate sensitivity/specificity of 0.643, which was statistically not significant (*p* = 0.226). Nonetheless, we suggest that this moderate sensitivity/specificity of 0.643 is acceptable for a notable association between total GHQ score (as the dependent variable) and HBA1c as test result (independent) variable (Figure 1).

## Discussion

Our study revealed that the non-diabetic individuals provided affirmative responses for most of the positive items (GHQ-1 to GHQ-6) compared to the diabetic and prediabetic individuals. This indicates that the mental health of the non-diabetic individuals was much better than that of the diabetic and prediabetic individuals. More specifically, the non-diabetic individuals reported that they could concentrate on whatever they were doing, and they were found to be reasonably happy compared to the prediabetic or the diabetic individuals. Similarly, we found that the non-diabetic individuals were also more capable of making decisions on specific tasks than their counterparts. Likewise, they outweighed in numbers in terms of being able to face problems the prediabetic and diabetic individuals.

In contrast, The GHQ-12 results showed that mental health was significantly worse among the diabetics and prediabetics than among the non-diabetics. More specifically, our study illustrated that the diabetic or the prediabetic individuals reported more on negative items (GHQ-7 to GHQ-12) with the tool assessing mental health. They seemed to have more negative attributes of mental health than the non-diabetics. In particular, the diabetic or the prediabetic individuals were higher in proportion in terms of losing sleep because of feeling worrisome or feeling unhappy or depressed about something. However, there were differences in proportion between the prediabetic and the diabetic individuals for some negative items. It was found that the prediabetic people outweighed the people with diabetes in terms of negative items in the GHQ. For instance, we found a significantly higher proportion of prediabetic individuals indicating feeling depressed or unhappy when compared to their diabetic counterparts. Likewise, losing confidence or thinking of oneself as a worthless person was reported more by the prediabetic than the diabetic individuals.

Furthermore, the findings of the adjusted analysis revealed that the patients with diabetes were more psychologically distressed than their counterparts. Likewise, with respect to gender, we found the women to be more psychologically distressed than the men. Unsurprisingly, the unemployed individuals were found to be more psychologically distressed than the employed individuals while adjusting for the sociodemographic variables.

As suggested by the GHQ-12 results, numerous previous studies point to poorer mental health among people with diabetes than among the general population. Some authors even advocate specific preventative treatments in such cases, given the importance they attach to the problem (Donmez et al., 2005). Our findings are consistent with majority of the studies in the existing literature. For instance, a case-control study conducted in Madrid city (Spain) shows that patients with diabetes in Madrid have poorer self-rated health and psychological wellbeing than subjects of the same age and gender without the disease (y Pena et al., 2010). Similarly, another study also found that diabetic patients were more likely to suffer from poor mental health and subclinical psychological distress than their counterparts of the same age and gender without diabetes (Jimenez-Garcia et al., 2012). Also, a cross-sectional study was conducted in the United Kingdom that conducted diagnostic interviews to screen for common mental health problems (Das-Munshi et al., 2007). The prevalence of mental health problems was 21.6% for diabetic individuals compared to 16.3% for those without diabetes (adjusted OR 1.5; 95% CI 1.1–2.2) (Das-Munshi et al., 2007). Likewise, another study conducted in the United States found that based on diagnostic codes of theVeterans HealthAdministration, the prevalence of any mental health problem among diabetes sufferers was 24.5% (Frayne et al., 2005).

“Fair/poor or very poor” self-perception of mental health is strongly related to suffering from diagnosed mental conditions or has a high result in the GHQ12 among individuals with diabetes in Saudi Arabia. Self-rated health status is a valuable gauge of the population's overall wellbeing and is a reliable measure of quality of life (Undén et al., 2008). Our results are aligned with most studies that have been conducted among diabetic individuals, found that two of the factors more obviously define diabetics' poorer quality of life or self-rated health include “suffering mental health problems” or “psychological distress” (Frayne et al., 2005; Das-Munshi et al., 2007; Undén et al., 2008).

This study's findings are consistent with other previous studies conducted across the globe. For example, the findings of a systematic review revealed that patients with type 2 diabetes (T2D) are more likely to be depressed, and that there is higher prevalence of depression among diabetic patients than among their non-diabetic counterparts (Ali et al., 2006). Furthermore, the same systematic review also found that diabetic women are more likely to be depressed than men (Ali et al., 2006). These results can be supported by the fact that patients with diabetes are more likely to be psychologically upset because of the nature of the disease. The existing evidence from cross-sectional and longitudinal studies finds an association between diabetes and negative psychological outcomes (Lin et al., 2010). Furthermore, such studies also reveal the relationship between glycemic control and negative psychological outcomes (Lustman et al., 2000). These findings are very germane to the context of Saudi Arabia from the perspective of taking preventive actions before such negative outcomes become causes of diabetic complications. This is because the existing evidence reveals that diabetic patients with poor psychological outcomes are more likely to develop complications from diabetes (Katon et al., 2010). This includes a myriad of complications such as diabetic retinopathy, neuropathy, nephropathy, diabetic foot, and microvascular complications (Lin et al., 2010). For instance, the findings of a meta-analysis demonstrated a strong association between depression and diabetes complications ranging from diabetic retinopathy to sexual dysfunction (Katon et al., 2010). Furthermore, the evidence also suggested that negative psychological outcomes such as depression can lead to dementia among diabetic individuals in the long run if untreated (Katon et al., 2010). In Taiwan, a population-based prospective cohort study followed up patients for up to 14 years. The study found that depression was associated with increased risk of macrovascular complications, and all-cause mortality in the diabetic cohort (Wu et al., 2020). The relationship between depression and prevalence of macrovascular complications is a complex multifactorial process in which the exact underlying mechanisms are still unknown, and only few studies have suggested some common processes. For instance, a study reported that depressive individuals usually follow an unhealthy lifestyle, smoke tobacco, lack physical activity, and take unhealthy diet (Deschênes et al., 2017). Mostly, depression is frequently accompanied with behavioral changes such us limited self-care and lack of adherence to medications. These behaviors in diabetic patients lead to poor glycemic control, which is eventually associated with higher risk of complications (Nouwen et al., 2019). Collectively, this evidence and the strong association between diabetes and negative psychological outcomes in our study call for urgent secondary prevention programs for diabetic people to prevent complications in the short and long run.

Likewise, our findings regarding the association between women and negative psychological outcomes based on GHQ score are analogous to the existing evidence, i.e., numerous studies have shown that women are more depressed than men (Albert, 2015). These findings of women being more affected by negative psychological outcomes are rooted in biological sex differences rather than external factors such as culture, dietary habits, level of education, and several other theoretically confounding social and economic reasons. However, it has also been found that this distorted ratio of negative psychological disorders prevails mainly at a young age and gets obscured in the older age (Cyranowski et al., 2000). The possible reason for explaining this finding could be due to the exposure of women to different reproductive phases of life (Albert and Newhouse, 2019). This is, in turn, correlated with hormonal changes, meaning that women are more affected by negative psychological outcomes because of hormonal changes during puberty, after becoming pregnant, and around menopause. This might suggest that hormonal changes could explicate the negative psychological outcomes in women (Albert and Newhouse, 2019). However, this was not ruled out in our study, but the existing evidence was sufficient to explain such findings. Irrespective of underlying mechanisms or causes of the negative outcomes, women, being vulnerable, are at higher risk of negative outcomes and should be paid more attention to avoid deterioration in their quality of life.

Lastly, in this study, the unemployed individuals were at greater risk of developing negative psychological outcomes than the employed individuals. This finding is comparable to other studies around the world. For example, findings from a meta-analysis showed that jobless individuals have a decreased level of psychological and physical wellbeing compared to employed individuals (McKee-Ryan et al., 2005). Such finding suggests that unemployed individuals might be defamed in society, which eventually affects their mental health status and reflects negative psychological outcomes (O'Donnell et al., 2015). This is further strengthened and supported by the contradictory evidence, which suggests that employed people are psychologically sound because of their satisfying life, fulfillment of their needs, and respect in society (Briner, 2000; Herriot, 2013). Furthermore, unemployment is related to marginalization, where unemployed people are disregarded or ignored in society and thereby not accepted in mainstream society, which is reflected in negative psychological outcomes (Kossen and McIlveen, 2018). Irrespective of underlying mechanisms for the association between unemployment and negative psychological outcomes, the government of Saudi Arabia needs to take urgent actions to help unemployed people by raising the job opportunities for them to protect them from experiencing negative psychological outcomes.

Given the findings of our study and their consistency with the existing evidence, healthcare providers need to be aware of the higher risk of psychological non-wellbeing and poor mental health among diabetic patients. We agree with previous studies that suggest screening for psychological distress, anxiety, and other mental health disorders multiple times per year among those diagnosed with diabetes and those who are borderline diabetics (Fisher et al., 2007). Concordant with previous studies that indicate that poorer mental health conditions may be under-diagnosed by clinicians, psychological problems are “least priority” compared to “overt” medical conditions in diabetic patients. Many physicians give less attention to poor mental health, because they feel that it is “expected” in patients with diabetes (Shao et al., 1997; Katon, 2008).

Similar to other studies, our study also has some limitations. One of the potential limitations of the research is that it has a cross-sectional study design. Thus, it could not assess the temporal relationship between self-perceived mental health and diabetes. We also did not evaluate the role of other factors such as age, gender, depression, lack of exercise, and obesity in the relationship between poorest self-perceived mental health and diabetes. These factors, therefore, need to be explored further in future epidemiological studies. Also, we did not record type 1 diabetes, duration of diabetes, the standard of care (antidiabetic agents or insulin) given to patients, and quality of care. All of these factors could impact patients' mental health and should, therefore, be included in any future investigations. This study has several strengths. Primarily, this study is conducted on a very large sample of both men and women from all the institutes of Al-Kharj by recruiting participants through a robust sampling technique. This allowed for us to recruit participants with different sociodemographic characteristics, thus enabling us to generalize our findings to the Saudi population.

## Conclusion

This study reveals that self-rated mental health and psychological wellbeing are substantially poorer among patients with diabetes or those who are prediabetics than those without diabetes. Thus, there is a need to screen diabetic patients for psychological wellbeing instead of waiting for an exact problem to be identified or worsening of psychological status. In addition, diagnosis, screening, and treatment need to be routinely incorporated into patients' regular care. Moreover, in light of the current findings, targeted programs and interventions need to be designed and implemented to cater to the mental health needs of diabetic and prediabetic individuals in Saudi Arabia. Furthermore, the government of Saudi Arabia needs to focus on diabetic, female, and unemployed individuals to prevent them from having negative psychological outcomes and long-term complications. Well-designed longitudinal studies are required in the future to study the cause-and-effect relationship between poor mental health and diabetes, mainly in Saudi Arabia. Moreover, further studies are needed to make causal inferences to examine the relationship between poor mental health and diabetes as well as important determinants of this relationship in the Saudi population.

## Data Availability Statement

The original contributions presented in the study are included in the article/supplementary material, further inquiries can be directed to the corresponding author.

## Ethics Statement

This study was reviewed and approved by the Institutional Review Board (IRB) of the College of Medicine, Prince Sattam Bin Abdulaziz University. The patients/participants provided their written and verbal informed consent to participate in this study.

## Author Contributions

All authors contributed to the writing of different manuscript sections and had access to the data. The final draft of the manuscript was read and approved by all authors.

## Conflict of Interest

The authors declare that the research was conducted in the absence of any commercial or financial relationships that could be construed as a potential conflict of interest.

## Publisher's Note

All claims expressed in this article are solely those of the authors and do not necessarily represent those of their affiliated organizations, or those of the publisher, the editors and the reviewers. Any product that may be evaluated in this article, or claim that may be made by its manufacturer, is not guaranteed or endorsed by the publisher.
